# Supplementary material for: Association between Healthy Dietary Patterns and Self-Reported Sleep Disturbances in Older Men: The ULSAM Study
Source: Nutrients. 2019 May 8;11(5):1029. doi: 10.3390/nu11051029 (PMC6566625; doi:10.3390/nu11051029)
Supplement: Supplementary file 1 [file nutrients-11-01029-s001.pdf]

Supplementary Table S1.

Table S1. Estimates of the parameters utilized to identify adequate reports of energy intake in the Uppsala Longitudinal Study of Adult Men (ULSAM).

**Variables**	**Total cohort (n=970)**	**Inadequate responders (n=451)**	**Adequate reporters (n=519)**
Physical activity level	1.6 ± 0.1	1.6 ± 0.1	1.6 ± 0.1
Energy intake (kJ/day)	7349 ± 1915	5920 ± 1275	8591 ± 1457
Basal metabolic rate (kJ/day)	6923 ± 566	7105 ± 608	6764 ± 473

Values are expressed as mean ± SD.
